# Carbon Nanohorns as Reaction Nanochambers – a Systematic Monte Carlo Study

**DOI:** 10.1038/s41598-018-33725-z

**Published:** 2018-10-18

**Authors:** Sylwester Furmaniak, Piotr A. Gauden, Andrzej Patrykiejew, Radosław Miśkiewicz, Piotr Kowalczyk

**Affiliations:** 1 Stanisław Staszic University of Applied Sciences in Piła, Podchorążych Street 10, 64-920 Piła, Poland; 2 Physicochemistry of Carbon Materials Research Group, Faculty of Chemistry, Nicolaus Copernicus University in Toruń, Gagarin Street 7, 87-100 Toruń, Poland; 3 Department for the Modelling of Physico-Chemical Processes, Faculty of Chemistry, Maria Curie Skłodowska University in Lublin, Gliniana Street 33, 20-031 Lublin, Poland; 4 Silesian University of Technology, Faculty of Organization and Management, Roosevelt Street 26, 44-100 Gliwice, Poland; 5 School of Engineering and Information Technology, Murdoch University, Murdoch, 6150 WA Australia

## Abstract

Carbon nanohorns (CNHs, one of the newest carbon allotropes) have been subjected to intensive experimental and theoretical studies due to their potential applications. One of such applications can be their use as reaction nanochambers. However, experimental studies on the reaction equilibria under confinement are extremely challenging since accurate measurements of the concentrations of reacting species in pores are a very hard task. So, the main ways to examine such phenomena are theoretical methods (e.g. the reactive Monte Carlo, RxMC). We have presented the first systematic RxMC study on the influence of the CNH’s geometric parameters (the apex angle, the diameter, and the length) on reaction equilibria, taking the nitrogen monoxide dimerisation as an example. All the investigated parameters significantly affect the reaction yield at low and moderate coverages. Short and narrow CNHs have been found to be preferred. However, the key factor influencing the reaction equilibria is the presence of a conical part. Energetics of interactions between the reacting molecules in this fragment of a nanohorn maximises the effects of confinement. In consequence, CNHs have the advantage over their nanotube counterparts of the same diameter. The obtained results have confirmed that CNHs can be considered as potential reaction nanochambers.

## Introduction

Carbon nanohorns (CNHs) are one the newest carbon allotropes. The discovery of single-walled carbon nanohorns was reported in 1999 by Iijima *et al*.^1^. CNHs spans a wide range of scientific interest. Terzyk *et al*.^2^ published a comprehensive review, covering the chronology of synthesis methods, properties and applications of these materials. The potential applications of CNHs can result from their unique microstructure. Carbon nanohorn samples synthesised with the use of different methods usually form globular aggregates – see ref.^2^ and the references therein. Such aggregates are composed of thousands of graphitic tubule-like systems, and hence CNHs are similar in structure to the single-walled carbon nanotubes (SWCNTs)^2–4^. However, the techniques to isolate individual CNHs from aggregates has also been proposed^5^. It has been accepted that the structure of CNHs consists of the pseudo-cylindrical tubular part and the conical tip^2,6–8^. Typically, the diameters and the lengths of CNHs range between 2 and 10 nm, and between 10 and 70 nm, respectively^2^. However, still narrower carbon nanohorns (with the internal pore radii varying in the range between 1.0 and 3.6 nm, and with the maximum at about 1.3 nm) have been also found^9^. The apex angles of the conical part have been experimentally observed to be close to 20°^10^.

Since the produced CNHs are closed, their internal space is unavailable. However, the procedures of controllable opening of nanohorns have been developed^11–13^. These methods are based on the chemical oxidation and thermal desorption of the formed oxygen functionalities^14–16^. Such modification leads to the creation of so-called nanowindows or nanogates, whose size can be controlled by the conditions of the process. The presence of nanogates makes the internal channels available to different molecules (which can be adsorbed inside CNHs). This option expands the range of CNHs applications. Experimental studies indicate that nanowindows are formed in two main locations: at the tips or at the sidewalls of CNHs^11,14,17–26^. However, the choice of conditions of the opening process makes it possible to control the places where the holes are preferentially created^11,14,17–19,24–26^. Moreover, the nanogates positioned at the tips of nanohorns can be thermally healed in the presence of Ar while the holes at sidewalls are more difficult to close^17,18^. So it is possible to prepare the samples of opened CNHs with undamaged conical parts.

In the literature reports describing possible applications of carbon nanohorns, the following major fields can be singled out: adsorption, catalysis, components applied for the preparation of electrodes (and generally application in power sources), nanomedicine and sensors^2,27–31^. One of such applications, closely related to the catalysis, can be the use of CNHs as reaction nanochambers. The internal channels are isolated nanovessels where different chemical reactions can take place. Moreover, one can expect that the effects of confinement affect the reaction equilibria^32^, and may potentially improve the reaction yield. Unfortunately, the experimental studies on chemical equibria in pores are extremely challenging, since accurate measurements of the concentrations of reacting species in pores are a very hard task^32–37^. Therefore, theoretical methods have become the main tools used to investigate such phenomena. One of the most convenient approaches used for this purpose is the reactive Monte Carlo technique (RxMC)^32,38–40^. Surprisingly, the number of reports based on the use of RxMC method to model reactions in pores is quite limited (see for example refs^32,37,41–54^) and this technique has not been yet used to study the effects of confinement in conical pores or inside CNHs. Hence, in the current study we have tried to fill this gap. Using the series of model CNHs consisting of the tubular and conical parts we have directly simulated reaction equilibria in their internal channels. We have studied how the confinement in pores of specific geometry affects the reaction yield. As the reference materials we have chosen single-walled carbon nanotubes. This choice is justified by the similarities in the structure. Some previous reports have suggested that SWCNTs can be treated as a good approximation of CNHs^55–58^. For example, our previous study^58^ showed that the differences between Ar adsorption isotherms inside CNHs and SWCNTs vanish when the length of nanohorn increases. This suggests that specific energetics of adsorption in conical parts have a negligible effect on some phenomena inside CNHs. However, this is not a general rule. Some other studies^59,60^ have clearly demonstrated the advantages of CNHs, due to the presence of conical tip, e.g. the equilibrium separation factor for adsorption of CO_2_/CH_4_ mixture has been found to always be higher inside CNHs than in SWCNTs of the same diameter (even for the longest nanohors)^61^.

We have chosen the nitrogen monoxide dimerisation as the simple model reaction:

1 $${\rm{NO}}+{\rm{NO}}\leftrightarrow {({\rm{NO}})}_{2}$$

The same reaction was considered also in other RxMC studies^43,44,48–50,53,54^. The nitric oxide dimerisation reaction is interesting for a number of reasons^43,44^. Firstly, it is an exothermic and thermodynamically driven reaction in which there is a decrease in the total number of moles. This reaction is important in atmospheric chemistry^62^ as well as in the human body, where NO regulates the blood pressure^63^. Moreover, predicting the effects of confinement on NO dimerisation is critical to pollution abatement since activated carbons are commonly used to remove nitrogen oxides from the autoexhaust and the industrial effluent gas streams^64^.

As we have mentioned above, the main aim of this study is to directly examine the effects of confinement inside CNHs on chemical equilbria and to check the possibility of their use as reaction nanochambers. The comparison of results for nanohorns and nanotubes have provided the answer to the question, whether the presence of conical parts in the CNH’s structure is important for the reaction yield. Besides, we have systematically studied the relationships between the geometric parameters of CNHs (apex angle, diameter and length) and the composition of reacting phase. Such information is crucial during the controlled synthesis of nanohorns for the purposes considered here.

## Results and Discussion

**Figure 1:**
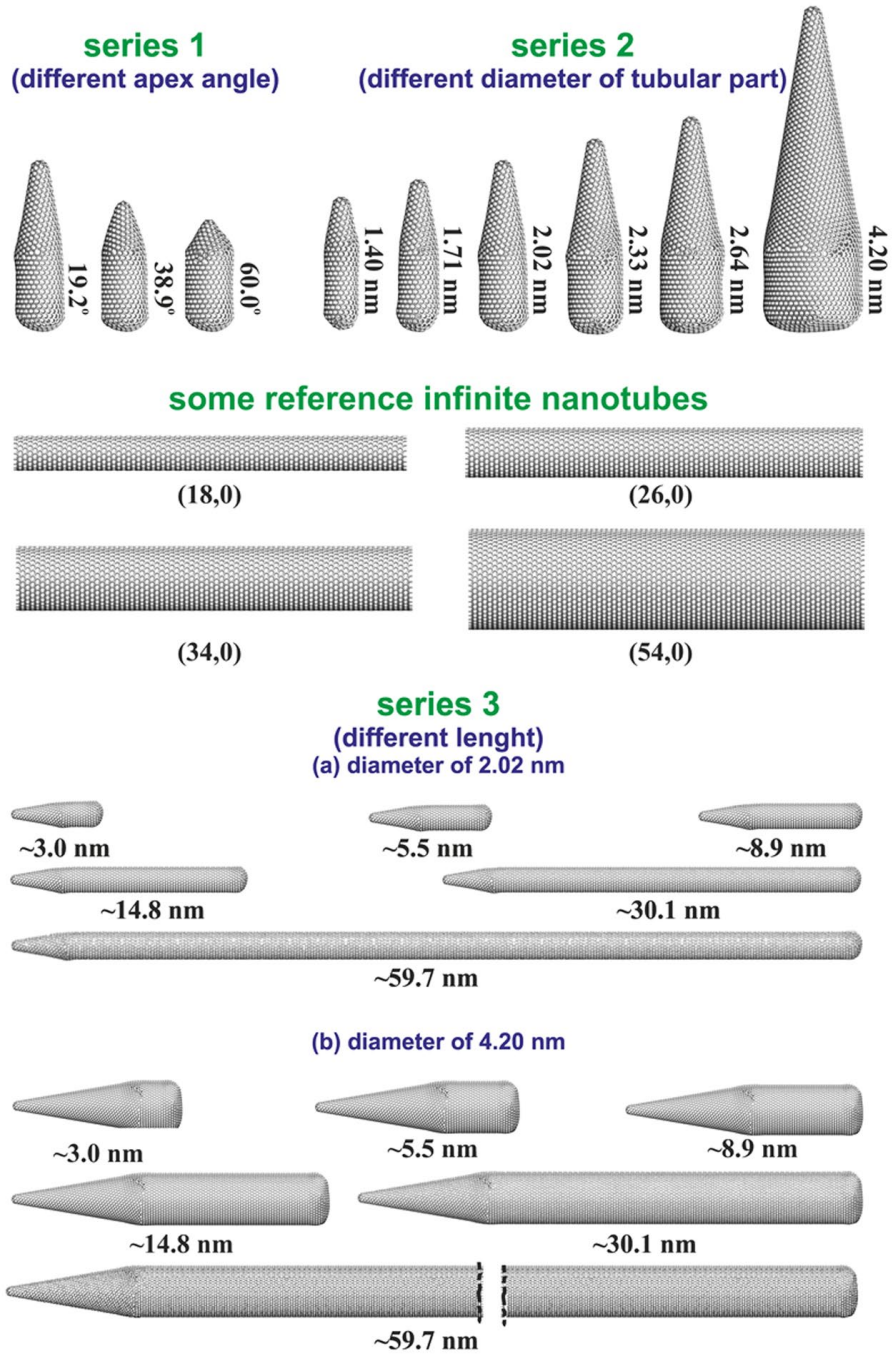
Schematic representation of all the studied model CNHs (divided into the series: 1, 2, 3a, and 3b) and selected reference SWCNTs. The CNHs in the series 3 are presented in another scales than other structures. It should be noted that the graphics collected in this figure have been created using the VMD program^68^ (http://www.ks.uiuc.edu/Research/vmd).

**Figure 2:**
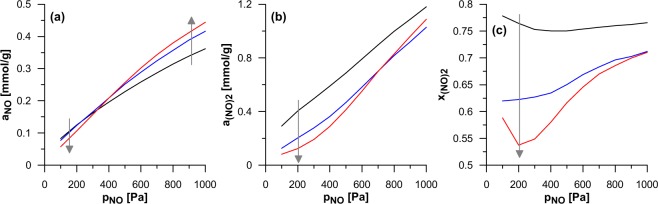
The influence of the CNH apex angle (series 1) on the composition of the reacting mixture. The amounts of NO (**a**), (NO)_2_ (**b**) and the mole fraction of the product – (NO)_2_ (**c**) are shown are shown versus the bulk NO pressure (*p*_NO_). The arrows show the direction of changes connected with the rise of the apex angle.

We begin the discussion of results by considering the effects of the CNH’s apex angle (series 1 in Fig. 1) on the reaction equilibria. Figure 2 compares the composition of the reacting phase inside these nanohorns. It is found that this parameter has a rather weak influence on the amount of the reactant (i.e. NO – Fig. 2a). For the lowest considered values of NO pressure, the CNH of the apex angle equal to 19.2° has some slight advantage over the others. This fact can be attributed to the energetics of adsorption in the conical part which is the highest for the lowest apex angle. Supplementary Animation 1 presents the changes in the filling of CNHs from the series 1. At the lowest values of *p*_NO_ the reacting molecules are present mainly close to the tip. As the pressure increases, the molecules appear also in other parts of CNHs, in particular near to the rounded bottom (where the energy of adsorption is higher than in the tubular part – for comparison see Fig. 5 in ref.^65^). In addition, for higher values of *p*_NO_ the amount of NO increases with the rise of the apex angle. This fact may be explained as the effect of the increasing contribution of adsorption in other parts of CNHs. However, the discussed picture may be also affected by the fact that the model nanohorn with the lowest apex angle consists of the highest number of carbon atoms^58^. This results in the reduction of NO amount calculated per the unit of CNH mass (see Eq. (2)). In this case, the amount of the product (i.e. (NO)_2_ – Fig. 2b) is the highest for the CNH of the lowest apex angle over the whole pressure range. The advantage of this nanohorn is also clearly seen in Supplementary Animation 1. It can also be associated with the energetic preferences in (NO)_2_ adsorption inside the conical part of CNH with the lowest apex angle. The described changes in the amounts of reacting molecules are reflected by the (NO)_2_ mole fraction (Fig. 2c). This quantity decreases when the apex angle increases. The highest reaction yield has been observed for the apex angle equal to 19.2°, i.e. similarly to the results observed for real nanohorns^10^.

**Figure 3:**
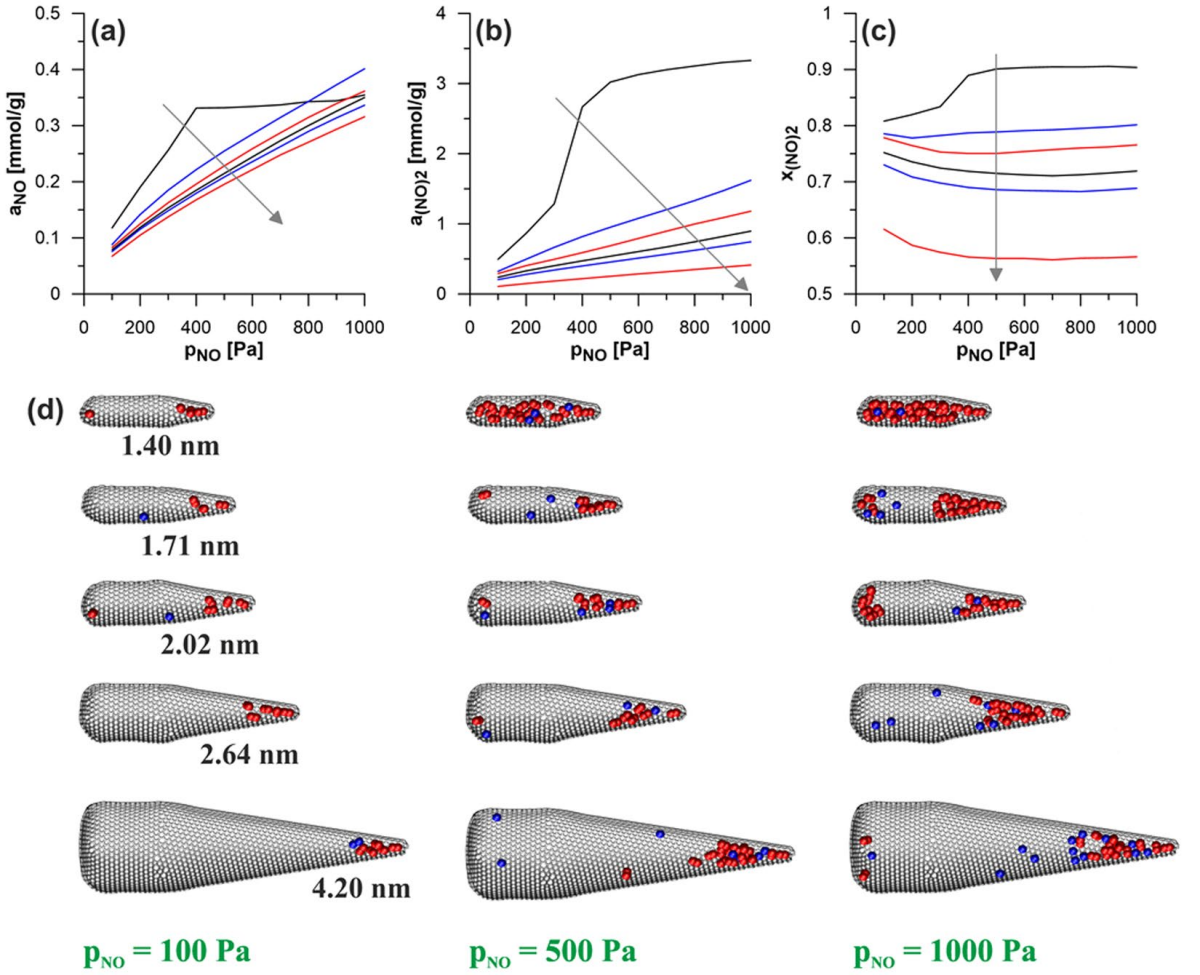
As in Fig. 2 but shows the influence of the CNH diameter (series 2) – the arrows show the direction of changes connected with the rise of the CNH diameter. In addition, the examples of equilibrium configurations are shown (**d**). The view after dividing the CNH along its axis into two parts. NO and (NO)_2_ molecules are marked by blue and red colour, respectively. It should be noted that the graphics collected in part (**d**) of this figure have been created using the VMD program^68^ (http://www.ks.uiuc.edu/Research/vmd).

The next parameter studied has been the diameter of tubular part (series 2 in Fig. 1). Figure 3a–c compares the composition of the reacting phase inside all the CNHs from this series. In addition, Fig. 3d and Supplementary Animation 2 show selected equilibrium configurations. The diameter of tubular part is the factor which has strong influence on the reaction yield. The increase of this parameter causes a gradual decrease of the amounts of the reactant as well as of the reaction product. Since this decrease is more pronounced in the case of (NO)_2_, also the mole fraction of this component decreases for wider CNHs. The highest reaction yield (*x*_(NO)2_ > 0.9) has been observed for the narrowest system (i.e. the nanohorn of the diameter equal to 1.40 nm). The effects of the changes in the NO bulk pressure should also be analysed. The rise in *p*_NO_ causes a gradual increase in the amount of both NO and (NO)_2_, regardless of the CNH’s diameter. However, in the narrowest nanohorn (of the diameter equal to 1.40 nm), the increase of NO and (NO)_2_ amounts is restricted. The amounts of reacting species hardly change when the pressure exceeds about 400 Pa. As one can see in Supplementary Animation 2, the reacting mixture fills the entire volume of the nanohorn above this value of pressure. Such condensation is connected with a step-like increase in the mole fraction of (NO)_2_ – Fig. 3c. Similar effects were reported and discussed also for the volume filling of pores with other geometries by dimerising NO mixture^43,44,48–50,53,54^. In the other studied systems such condensation has not been observed, under similar conditions. However, the reaction yield in these CNHs has also been found to be high in comparison to the same reaction occurring in the gaseous phase (in the studied range of pressure the mole fraction of (NO)_2_ for the reaction in the bulk increases linearly from 3.02 × 10^−6^ up to 3.02 × 10^−5^). Similarly as in the case of series 1, we have observed that high values of *x*_(NO)2_ are the consequence of the energetic preferences in adsorption of product molecules in conical parts. The majority of molecules (Fig. 3d and Supplementary Animation 2) is present in this fragment. As the pressure increases, the cluster of reacting molecules grows and occupies wider parts of the cone. A careful analysis of the influence of the pressure increase on the product mole fraction (Fig. 3c) may be surprising. Since in the studied reaction (cf. Eq. (1)) the mole volume for the product is lower than for the substrate (one molecules is formed from two), one can except that the rise in pressure should cause the increase of the reaction yield. Such behaviour has been observed for the reaction in infinite nanotubes of the same diameters as the nanonohorns in the series 2 – the effects of SWCNT’s diameter on the composition of the reacting phase are shown in Supplementary Fig. S1. However, in the case of the CNHs from the discussed series (with the exception of the narrowest one) the highest yield has been observed for the lowest value of *p*_NO_. The explanation of this difference in comparison to SWCNTs of energetically homogeneous surfaces, is based on the different structure of CNHs. At initial stages (low pressures) the reacting mixture is adsorbed mainly near the tip of conical part (Supplementary Animation 2). The rise in *p*_NO_ causes that the molecules appear also in other parts on CNH (wider fragments of the cone, the tubular part and at the bottom). Since the energetic preferences of (NO)_2_ adsorption over the adsorption of NO molecules at these places are weaker than in the tip, the reaction yield, averaged over the whole volume of CNH, decreases.

**Figure 4:**
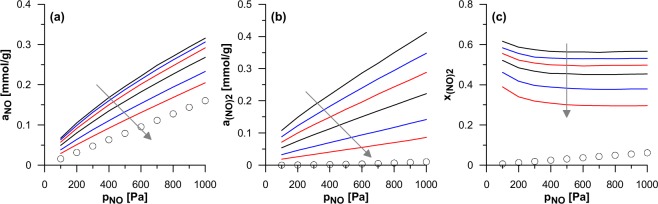
As in Fig. 2 but shows the influence of the CNH length (series 3b, i.e. CNHs with diameter of 4.20 nm). In addition, the open circles show the data for the infinite SWCNT of the same diameter. The arrows show the direction of changes connected with the rise of the length.

The last studied parameter has been the length of the tubular part (series 3a and 3b in Fig. 1). Supplementary Fig. S2 (series 3a) and Fig. 4 (series 3b) present the effects of the model nanohorns lengthening. For both subseries, also the data for infinite nanotubes of the same diameter have been shown as the reference. The amounts of the reactant (NO) in CNHs are only moderately higher than in SWCNTs. However, there are dramatic differences in the product ((NO)_2_) amounts, which have been found to be much higher in nanohorns. This is reflected by high mole fractions of dimers. Of course, the rise in the length is connected with the reduction of the reaction yield. The longer tubular part causes that the probability of the molecules presence in this part is higher (as it is shown in Supplementary Animation 3). In consequence (similarly as it has been discussed above), the average reaction yield is reduced. However, it is still larger than inside nanotubes – even for the longest studied nanohorns. It should be noted that the size of the last CNHs in the series 3b is comparable with the experimentally observed^10^.

**Figure 5:**
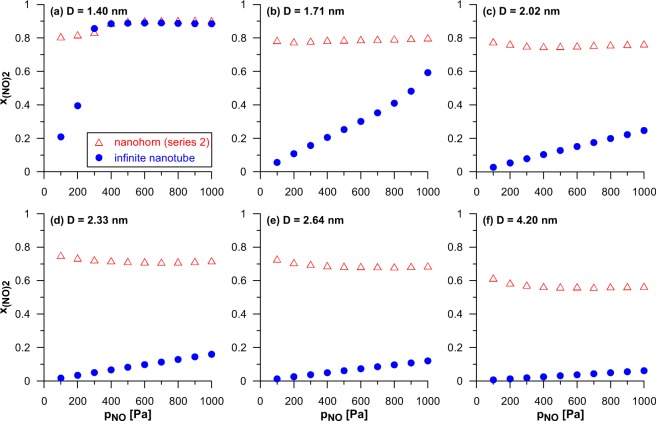
The comparison of the product mole fractions (*x*_(NO)2_) inside the CNHs of the series 2 and the SWCNTs of the same diameter (the indicated values).

Finally, Fig. 5 compares the product mole fractions inside nanohorns from the series 2 and their nanotube counterparts. In addition, Supplementary Animation 4 shows configurations of molecules inside the selected CNHs and SWHCNTs of the same diameter. In the case of the narrowest systems (*D* = 1.40 nm – Fig. 5a) the CNH has the advantage over the SWCNT only in the low pressure range. For the higher values of *p*_NO_ (where the above-mentioned volume filling occurs – Supplementary Animation 4) the reaction yield is practically the same. Such behaviour indicates that the effects of capillary condensation dominate over the differences in adsorption energy between the nanohorn and the nanotube. For the other studied diameters the yield of the dimerisation reaction inside CNHs is higher than inside SWCNTs in the whole range of considered here pressures. Moreover, the disproportions between nanohorns and nanotubes increase as their diameter becomes higher. The presence of the conical part in the structure of CNH (where the cluster of reacting molecules is formed and the adsorption of (NO)_2_ molecules is energetically favoured regardless of the size of other parts) partially compensates the reduction of reaction yield when the tubular part becomes wider. The observed behaviour can be also interpreted in the context of the Le Chatelier’s principle. Since the molar volume of the product is smaller than for the equivalent amount of the reactant, the reacting mixture prefers the restricted volume of the conical parts, where the molecules are moved. The accumulation of molecules in the small volume leads to the increase in the reaction yield in comparison to the bulk phase and to the wide homogeneous channels in CNTs.

**Figure 6:**
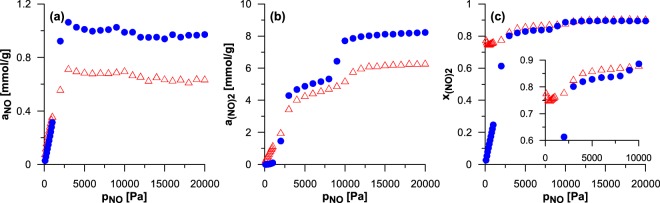
The comparison of the composition of the mixture reacting inside the CNH from the series 2 of diameter equal to 2.02 nm (triangles) and inside its nanotube counterpart (circles).

The above-discussed regularities concern the low values of the NO bulk pressure which result in low or moderate coverage of the surface of the studied nanomaterials (expect for the narrowest ones). Such conditions have been deliberately chosen since low surface coverage is typical for many catalytic processes with gaseous reactants. Moreover, sub-monolayer adsorption is the most sensitive for the differences resulting from the specific nanostructure of the studied materials. In fact, the results for the narrowest nanohorn in the series 2 have revealed that the advantage of this CNH over its nanotube counterpart vanishes when the reacting mixture condenses in their whole volume. In order to carefully examine such effects we have performed some additional simulations for higher values of the NO pressure for the one nanohorn from the series 2 of the diameter equal to 2.02 nm and for the nanotube of the same size. The obtained results are collected in Fig. 6. The changes in the amount of (NO)_2_ (Fig. 6b) indicates two-step mechanism of nanospace filling. Firstly the monolayer is formed (for *p*_NO_ ca. 4000 Pa) and next the remaining volume is filled (for *p*_NO_ ca. 10000 Pa). Here, it should be noted that the difference in the maximum values of *a*_(NO)2_ (and also of *a*_NO_) between the systems reflects the difference in the accessible volume per the unit of carbon mass (this is much smaller for CNH). The amount of NO in both nanostructures (Fig. 6a) increases only before the monolayer is filled. Then, even some decrease is observed (it results from the blocking of adsorption sites by the preferentially adsorbed (NO)_2_ molecules). This behaviour is related to the changes in the product mole fraction as the pressure rises (Fig. 6c). In the case of CNH the initial decrease in the yield of reaction (see also Fig. 3c) changes into the increase. The significant change in *x*_(NO)2_ (from ca. 0.75 up to ca. 0.89) is connected with the monolayer formation (the further rise connected with the condensation in the whole volume is only slight). In contrast, the formation of monolayer on the surface of nanotube causes the colossal increase in the product mole fraction. At this stage *x*_(NO)2_ becomes only slightly lower than for CNH. The differences in the reaction yield between nanotube and nanohorn vanish as the whole volume is filled (similarly as in the case of the narrowest system in the series 2). The results shown in Fig. 6 indicate that the increase in the coverage (the monolayer creation or capillary condensation) leads to the some significant rise in the yield of the studied reaction (connected with increasing energy of fluid-fluid interactions). However, the advantage of CNH over the SWCNT vanishes simultaneously. This is not surprising since the volume of conical part is only a small part of the whole accessible volume. Thus, the main factor responsible for the composition of the reacting phase after its condensation is the size of the dominant tubular part. The similar behaviour (i.e. the lack of differences toward the nanotube after volume filling) can be expected also for the CNHs of other sizes.

Summing up, we have presented the results of the first systematic Monte Carlo study on the influence of the carbon nanohorns geometric parameters on the reaction equilibria inside, taking the nitrogen monoxide dimerisation reaction as an example. The key-factor responsible for the high reaction yield at low and moderate coverages is the presence of conical part. The apex angle equal to 19.2° (similar to experimentally observed) is related to the highest amount of the product. The increase in the other two parameters (i.e. the diameter and the length) causes a reduction of (NO)_2_ mole fraction. So, the short and narrow CNHs seem to be the best reaction nanochambers. Regardless of the geometrical parameters, carbon nanohorns have the advantage over the nanotubes of the same diameter – even for the large sizes, similar to the real systems (this advantage vanishes at higher pressures when the capillary condensation occurs, however, the volume filling is connected with the highest reaction yield). The obtained results suggest that CNHs can be considered as potential reaction nanochambers. Due to energetics of interactions between reacting molecules and the walls of conical parts one can except to maximize the effects of condiment. In the context of our previous study^66^, the reaction yield, leading to the product(s) whose energy of interactions with carbon is higher than for reactant(s), should be improved in comparison to the bulk phase.

## Methods

We have used the three series of the model CNHs generated and described previously^58,61^. They are shown in Fig. 1. The series 1 includes CNHs of different apex angle, and the same other geometric parameters, i.e. the diameter and the length of tubular parts equal to 2.02 nm and 3.0 nm, respectively. The series 2 includes CNHs of different diameter of tubular part and the same other parameters (the apex angle: 19.2° and the length of the tubular part: 3.0 nm). The series 3 includes CNHs of different length and it is consisted of two subseries differing in the diameter of the tubular parts (i.e. 3a: 2.02 and 3b: 4.20 nm) and of the same apex angle (19.2°). As reference materials we have chosen infinite SWCNHs of the same diameter as the studied CNHs, i.e. (18, 0), (22, 0), (26, 0), (30, 0), (34, 0), and (54, 0). The fragments of the SWCNHs (with the length of 16.92 nm) have been modelled with periodic boundary conditions along their axes.

We have modelled the reaction equilibrium of nitrogen monoxide dimerisation (Eq. (1)) inside all the CNHs (and SWCNTs) using the RxMC method, at *T* = 125 K. We have implemented the simulation scheme proposed by us previously^66^. This approach assumes the adsorption equilibrium of reactants molecules (NO) with the gaseous phase. We have chosen the bulk pressure of NO (*p*_NO_) from the range 100–1000 Pa, in order to avoid volume filling of the wider CNHs by the reacting mixture. Effects of condensation of the studied reacting systems in pores were sufficiently studied in other works^43,44,48–50,53,54^. Besides, the reaction equilibria have been simulated also at higher values of *p*_NO_ (up to 20 000 Pa) in the case of the one CNH from the series 2 of diameter equal to 2.02 nm and its nanotube counterpart. NO and (NO)_2_ molecules have been modelled as single and two identical Lennard-Jones centres (with bond length equal to 0.2174 nm), respectively, according to the force field proposed by Kohler *et al*.^67^. The similar simple models have also been successfully used in other RxMC studies^43,44,48–50,53,54^. In these works the value of collision diameter for the centres in (NO)_2_ dimers in the original Kohler *et al*. approach^67^ was modified and assumed to be equal to the collision diameter for molecules of NO monomers. Some other simulation details are described in the Supplementary Information.

The average numbers of molecules in pores ($\langle {\rm{NO}}\rangle$ and $\langle {({\rm{NO}})}_{2}\rangle$), determined during simulations, have been used to calculate the amounts of the reacting species per the unit of adsorbent mass:

2 $${a}_{{\rm{NO}}}=\frac{\langle {\rm{NO}}\rangle }{{N}_{{\rm{C}}}{M}_{{\rm{C}}}}$$

3 $${a}_{({\rm{NO}})2}=\frac{\langle {({\rm{NO}})}_{2}\rangle }{{N}_{{\rm{C}}}{M}_{{\rm{C}}}}$$

where *N*_C_ and *M*_C_ are the number of carbon atoms forming nanohorn (nanotube) and the carbon molar mass, respectively. We have also calculated the mole fraction of the product ((NO)_2_):

4 $${x}_{({\rm{NO}})2}=\frac{\langle {({\rm{NO}})}_{2}\rangle }{\langle {\rm{NO}}\rangle +\langle {({\rm{NO}})}_{2}\rangle }$$

## Electronic supplementary material

Supplementary information

Supplementary animation 1

Supplementary animation 2

Supplementary animation 3

Supplementary animation 4

## Data Availability

All the data generated or analysed during this study are included in this published article and its Supplementary Information files.
