# Supplementary material for: Carbon Nanohorns as Reaction Nanochambers – a Systematic Monte Carlo Study
Source: Sci Rep. 2018 Oct 18;8:15407. doi: 10.1038/s41598-018-33725-z (PMC6194008; doi:10.1038/s41598-018-33725-z)
Supplement: Supplementary file 1 — Supplementary information [file 41598_2018_33725_MOESM1_ESM.pdf]

Supplementary information

for

Carbon Nanohorns as Reaction Nanochambers ï a Systematic Monte Carlo Study

Sylwester Furmaniak, Piotr A. Gauden, Andrzej Patrykiejew,
Radosğaw MiŜkiewicz, Piotr Kowalczyk

number of pages 4

number of tables 1

number of figures 2

Simulation details

The applied methodology of RxMC simulations[1] assumes the adsorption equilibrium of the reactants in pores and in the bulk phase. This equilibrium has been realised by the use of the trial moves typical for the grand canonical Monte Carlo simulations (GCMC), i.e. creation and destruction of NO molecules at random places in the simulation box. Since we have considered low values of NO pressure (p_{NO}) in the gaseous phase, the NO activity has been calculated as for an ideal gas. For each value of p_{NO} the simulation run has consisted of 1×10^7 cycles. During one cycle, 5000 iterations have been preformed. Each iteration has been a single attempt to change the system state via a randomly chosen trial move (the creation or the annihilation of a molecule of the reactant and the displacement (and/or rotation in the case of $(NO)_2$) of randomly chosen molecule (both the reactant as well as the product)). The probability of the use of each of these perturbations has been the same (i.e. 1/3). In addition, randomly chosen attempts of a forward or backward reaction step has been performed during iterations chosen with the probability equal to 1/500. The criteria of acceptation for the trial moves has been the same as listed in Table S1 in Ref. 1. Implementation of this formalism for reaction steps for the studied NO dimerisation reaction can be found also in Ref. 2. The first 4×10^6 cycles have been used to equilibrate the system, and the data have been collected during the next 6×10^6 cycles.

Since the used models of NO and $(NO)_2$ molecules includes only LJ centres[3], the energy of the system has been calculated as the sum of LJ interactions between pairs of the centres (centres in reacting molecules and carbon atoms in CNHs and SWCNHs). The values of LJ potential for different kinds of centres are collected in Table S1. The interactions between all the pairs have been cut at the distance equal to 5 × ů$_{ij}$.

The value of reaction quotient (ũ = 5.206×10^{-2} nm^3) for the nitrogen dimerisation at the studied temperature (T = 125 K) has been taken from Ref. 4. This value of ũ corresponds to the standard free-energy change (φG°) equal to 6.032 kJ/mol ï see Ref. 4.

Description of the supplementary animations

Examples of equilibrium configurations for all the studied values of NO pressure in the bulk phase (p_{NO}). The views after dividing the CNH or SWCNT along its axis into two parts. NO and $(NO)_2$ molecules are marked by blue and red colour, respectively.

Animation 1: All three CNHs from the series 1 (different apex angles as shown).

Animation 2: Selected CNHs from the series 2 (different diameters as shown).

Animation 3: Selected CNHs from the series 3b (different lengths as shown) and, in addition, the infinite SWCNH of the same diameter (4.20 nm), i.e. (54,0).

Animation 4: Comparison of the selected CNHs from the series 2 and infinite SWCNHs of the same diameter (as shown).

It should be noted that the graphics in all the animations have been created using the VMD program[6] (http://www.ks.uiuc.edu/Research/vmd).

References

1. Furmaniak, S., Gauden, P. A., Kowalczyk, P. & Patrykiejew, A. Monte Carlo study of chemical reaction equilibria in pores of activated carbons. *RSC Adv.* **7**, 53667-53679 (2017).
2. Lísal, M., Brennan, J. K. & Smith, W. R. Chemical reaction equilibrium in nanoporous materials: NO dimerization reaction in carbon slit nanopores. *J. Chem. Phys.* **124**, 064712 (2006).
3. Kohler, F., Bohn, M., Fisher, J. & Zimmermann, R. The excess properties of nitric oxid mixtures. *Monatsh. Chem.* **118**, 169-182 (1987).
4. Lísal, M., Cosoli, P., Smith, W. R., Jain, S. K. & Gubbins, K. E. Molecular-level simulations of chemical reaction equilibrium for nitric oxide dimerization reaction in disordered nanoporous carbons. *Fluid Phase Equilib.* **272**, 18-31 (2008).
5. Steele, W.A. The interaction of gases with solid surfaces. Pergamon: Oxford, 1974.
6. Humphrey, W., Dalke, A. & Schulten, K. VMD ï visual molecular dynamics. *J. Mol. Graphics* **14**, 33-38, (1996).

Table S1

The values of LJ parameters applied during simulations.

	centre	ů [nm]	$Ůk_B$ [K]	Reference
NO	NO*	0.31715	125.0	3
$(NO)_2$	NO*	0.35060	125.0	
CNH/SWCNT	C	0.34000	28.0	5

* the united atom

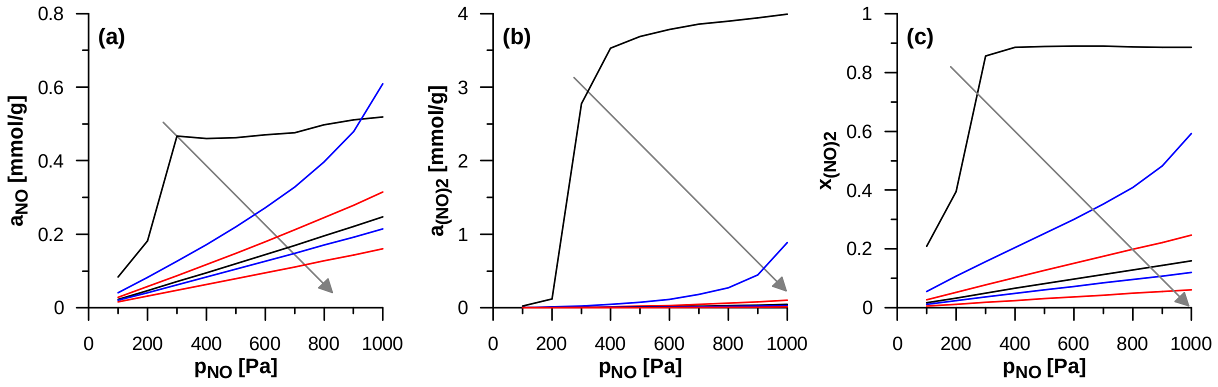

Figure S1. The influence of SWCNT diameter on the composition of the reacting mixture. The amounts of NO (a), $(NO)_2$ (b) and the product mole fraction ï $(NO)_2$ (c) are shown versus the bulk NO pressure. The arrows show the direction of changes connected with the diameter increase

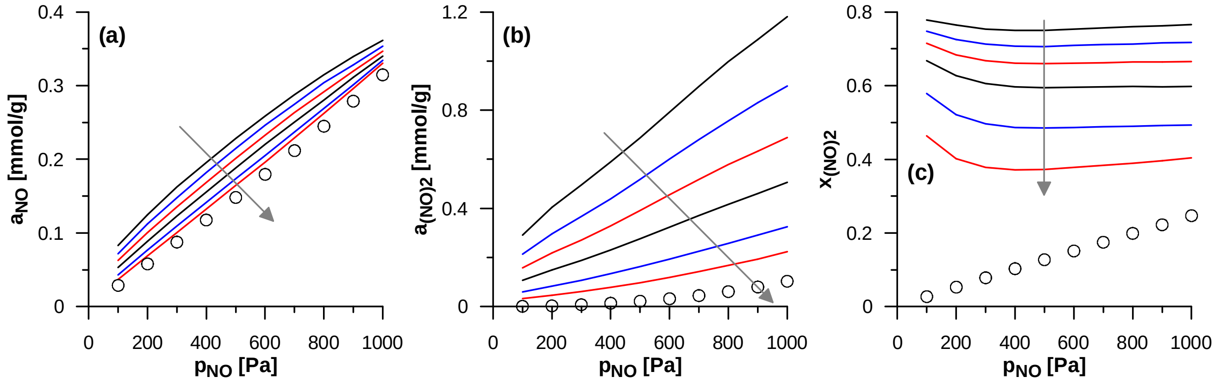

Figure S2. Similarly as in Figure S1 but shows the influence of the CNH length (series 3a, i.e. CNHs with the diameter of 2.02 nm). In addition, the open circles show the data for the infinite SWCNT of the same diameter. The arrows show the direction of changes connected with the increase of the length.
